# Getting the right fit: Convergence between preferred and perceived involvement in treatment decision making among medical oncology outpatients

**DOI:** 10.1002/hsr2.101

**Published:** 2018-11-06

**Authors:** Elise Mansfield, Jamie Bryant, Mariko Carey, Heidi Turon, Frans Henskens, Alice Grady

**Affiliations:** ^1^ Health Behaviour Research Collaborative, School of Medicine and Public Health, Faculty of Health and Medicine University of Newcastle Callaghan Australia; ^2^ Priority Research Centre for Health Behaviour University of Newcastle Callaghan Australia; ^3^ Public Health, Hunter Medical Research Institute New Lambton Heights Australia; ^4^ Population Health, Hunter New England Local Health District Wallsend Australia

**Keywords:** cancer, patient preferences, patient‐centered care, treatment decision making

## Abstract

**Background and Aims:**

While cancer patients' preferences for their level of involvement in treatment decision making (TDM) vary, previous research indicates a large proportion of patients are not experiencing TDM that meets their preferences. Evidence is needed to identify the characteristics of cancer patients who are less likely to report experiencing their preferred level of involvement in TDM, so that appropriate decision‐making support can be provided to them. We examined in a sample of medical oncology outpatients (1) the level of agreement between preferred and perceived involvement in TDM and (2) demographic, psychological, disease, and treatment characteristics associated with having unmet preferences for involvement in TDM.

**Methods and Results:**

Cancer patients from three medical oncology treatment centers in Australia completed surveys assessing demographic, disease and treatment variables, psychological distress, and preferred and perceived involvement in TDM. Data were collected between February 2013 and December 2014. Factors associated with having unmet TDM preferences were examined using logistic regression. There were 355 patients included in the analysis (75% response rate). The mean age (±SD) of the participants was 61 (±12), and 45% were male. Overall, 60% of participants reported that their preferences for involvement in TDM were met. No demographic, psychological, disease, or treatment characteristics were significantly associated with an increased probability of not having TDM preferences met.

**Conclusions:**

In line with previous research, a large proportion (40%) of patients reported TDM experiences that were not in alignment with their preferences. Future research should explore additional characteristics that are associated with a lower likelihood of having TDM preferences met.

## 1 INTRODUCTION

Cancer treatment decision making (TDM) often involves treatment options that have similar survival outcomes.^1^ Treatment decisions can depend on trade‐offs between possible risks and side effects of treatment,^2^, ^3^ the circumstances and life stage of the patient,^4^ and potential impacts on quality of life.^3^ As such, the active engagement of patients in TDM, often termed shared decision making, is considered to be critical to ensuring patient‐centered care.^5^ The involvement of cancer patients in TDM has been shown to improve outcomes such as physical function^6^ and quality of life.^7^ It has been argued that to achieve shared TDM, patients should take a more assertive and active role^8^ in decisions.

While a great deal of research effort has been invested in finding ways to support cancer patients to become more involved in making treatment decisions, not all patients prefer an active role in this process.^9^, ^10^ Preferences for participation in cancer TDM have been shown to vary by a range of factors, including age,^11^, ^12^ ethnicity,^13^ and education.^14^ Giving patients a more active role when this is unwanted can result in negative outcomes such as low satisfaction and decisional regret.^15^ In contrast, agreement between preferred and perceived level of involvement in decision making is associated with satisfaction with decision making^16^, ^17^, ^18^ and lower decisional conflict.^19^ However, current literature suggests that preferences for involvement in TDM are not met (ie, do not match their preferred level of involvement) for a large proportion of cancer patients.^9^, ^10^ Systematic reviews conducted in 2010 and 2014 showed mean congruence between preferred and perceived involvement in cancer TDM to be only 61% and 58%, respectively, indicating that preferences for involvement in TDM are not met for many patients.^9^, ^10^

Given the negative outcomes associated with failing to meet preferences for involvement in TDM, identifying patients who are less likely to have their preferences met can assist in ensuring that these patients receive additional support to make decisions.^9^ Few studies have explored which characteristics are associated with a lack of concordance between preferred and perceived TDM.^9^ Only a limited range of possible associations have been explored (eg, education^20^ and psychological distress^21^, ^22^), with mixed findings. Other characteristics associated with lower likelihood of preferences being met may include cancer site, given evidence indicating wide variation across patients with different cancer types in preferences for involvement in TDM, and time since diagnosis, given experience gained in dealing with health systems and expressing preferences. More research is needed to identify the characteristics of patients who are less likely to have their preferences met.

This study aimed to examine, in a sample of medical oncology outpatients, (1) the level of agreement between preferred and perceived involvement in TDM and (2) demographic, psychological, disease, and treatment characteristics associated with having unmet preferences for involvement in TDM.

## 2 METHODS

### 2.1 Design and setting

This was a cross‐sectional study undertaken in a convenience sample of three medical oncology treatment centers located in New South Wales, Queensland, and Tasmania, Australia. Two centers were located in metropolitan areas, and one was located in a rural area. Treatment centers were eligible if they provided care to at least 400 patients per year.

### 2.2 Sample

Eligible patients were those who had a confirmed cancer diagnosis, were attending at least their second outpatient appointment, were aged at least 18 years, were able to read English, and had capacity to provide consent.

### 2.3 Procedure

Potentially eligible patients were identified from clinic lists and approached by a trained research assistant in the waiting room prior to their appointment. Consenting participants were provided with an initial pen and paper survey. A follow‐up survey was mailed to participants approximately 1 month later. Reminder letters were sent to nonresponders after approximately 3 and 6 weeks of nonresponse. Procedures were approved by the Cancer Institute New South Wales, each hospital's ethics committee, and the University of Newcastle. All participants provided their informed consent. Data were collected between February 2013 and December 2014.

### 2.4 Measures

The initial survey included the following.

*Demographic variables*: Participants reported their age, sex, education, marital status, living situation, and whether they held private health insurance or a concession card.

*Disease and treatment variables*: Participants self‐reported their cancer type, time since diagnosis, and whether they had received surgery, radiotherapy, chemotherapy, or other treatments.

*Psychological distress*: The Hospital Anxiety and Distress Scale (HADS)^23^ was used to measure distress. Respondents are asked to rate the extent to which they have experienced 14 symptoms over the past week. Each item is scored 0 to 3, with higher scores indicating more severe distress. Items are grouped into anxiety and depression subscales (maximum score 21). A score of at least 8 on each subscale was used to indicate possible cases of anxiety and/or depression.^23^ The HADS has been shown to have adequate construct and discriminant validity in populations of patients with cancer and adequate internal consistency.^24^

The follow‐up survey included the following.

*Preferred and perceived involvement in TDM*: Participants reported when their last important decision about their cancer treatment was made (in days, weeks, or months) and how involved they were in making that decision. Participants also reported how involved they would like to be in making important treatment decisions (please see the Supporting Information for survey items). Response options were adapted from the Control Preferences Scale.^25^ Given that the original Control Preferences Scale was a card‐sorting task, this was not feasible to include in a pen and paper survey. Similar adapted versions of the scale have been used in other studies.^26^, ^27^, ^28^

### 2.5 Analysis

Analyses were completed using SAS v9.4 (SAS Institute, Cary, North Carolina). Analyses were conducted on the available data for each aim. Frequencies and percentages of patients' preferred and perceived involvement in TDM were calculated. Weighted kappa statistics (using Cicchetti‐Allison agreement rates) were used to indicate the level of agreement between preferred and perceived involvement in TDM for nonmissing cases.

Logistic regression modelling with “preference not met” as the outcome was performed for demographic, cancer and treatment characteristics, and HADS scores (adjusted for age). Unadjusted and adjusted odds ratios, Wald 95% confidence intervals (CIs), and *P* values were calculated. Inclusion of variables in adjusted analyses was decided by content experts, applying forward selection, with a limit on the number of parameters estimated to approximately one per 10 patients in the smallest outcome group. Logistic regression analysis was limited to only those participants who had complete data for the variables of interest (n = 279). Our sample of 279 patients had 80% power to detect an odds ratio of at least 1.9 assuming that one group has an exposure probability of 0.5.

## 3 RESULTS

### 3.1 Sample

A total of 823 patients were screened for eligibility to participate. Of these, 698 patients were eligible and 612 (87.7%) consented to participate, with 473 (77.3%) participants completing the initial survey. Of these, 355 participants (75% response rate) completed the follow‐up survey and were included in this analysis. There were no differences in age and sex for nonconsenters as compared with participants who completed the follow‐up survey (both *P* > 0.05). Table 1 shows the characteristics of the sample.

**Table 1:** Sample demographic, psychological, disease, and treatment characteristics (N = 355)^a^

Variable		Number (%)
Age, y	18‐40	21 (6%)
	41‐60	140 (40%)
	61‐79	179 (51%)
	80+	11 (3%)
Sex	Male	159 (45%)
Marital status	Married or partner	243 (69%)
	Single, divorced, separated, or widowed	110 (31%)
Education	High school or below	151 (43%)
	Vocational training, university, or other	199 (57%)
Health insured	Yes	163 (46%)
Concession card	Yes	189 (54%)
Living arrangement	With others	279 (79%)
	Alone	74 (21%)
Cancer type	Hematology	13 (3.7%)
	Breast	99 (28%)
	Colorectal	79 (23%)
	Prostate	21 (6.0%)
	Lung	20 (5.7%)
	Melanoma	12 (3.4%)
	More than one type or other	105 (30%)
Time since diagnosis, mo	12 or less	182 (52%)
	13 to 24	51 (14%)
	More than 24	119 (34%)
Treatment received^b^	Surgery	251 (71%)
	Chemotherapy	299 (86%)
	Radiotherapy	173 (52%)
	Other	16 (4.8%)
Number of different types of treatment received	1	2 (0.6%)
	2	82 (23%)
	3	117 (33%)
	4	115 (33%)
	5	31 (8.8%)
	6	5 (1.4%)
Anxiety	Yes	82 (23%)
Depression	Yes	63 (18%)
Study site	1	108 (30%)
	2	120 (34%)
	3	127 (36%)

a Not all variables sum to 355 because of missing data.

b Participants could select more than one option.

### 3.2 Preferred vs perceived involvement in TDM

The majority (70%) of patients made their last important treatment decision within the 6 months prior to being surveyed. Table 2 shows participants' preferred and perceived roles in their last important treatment decision. An approximately equal proportion of patients preferred making the decision themselves after seriously considering the doctor's opinion (34%) or shared responsibility for TDM (32%). However, when considering their last important treatment decision, fewer patients perceived that they had these levels of involvement in TDM (26% and 27%, respectively).

**Table 2:** Proportions of patients reporting each type of preferred and perceived involvement in treatment decision making (TDM) (n = 355)

Response Option	Type of Decision Making	Preferred Involvement, N (%)	Perceived Involvement, N (%)
Patient makes decision	Active	5 (1%)	23 (7%)
Patient makes decision after seriously considering doctor's opinion	Active	120 (34%)	89 (26%)
Shared responsibility	Shared	110 (32%)	93 (27%)
Doctor makes decision after seriously considering patient's opinion	Passive	62 (18%)	42 (12%)
Doctor makes decision	Passive	51 (15%)	95 (28%)
Missing		7	13

One‐third of the patients reported that they preferred a more passive role, including the doctor making the decision after considering the patients opinion (18%) or the doctor making the decision alone (15%). However, while only 15% of patients reported that they preferred for the doctor to make the decision, almost twice the number of patients (28%) perceived that this type of TDM had occurred for their last important decision.

Table 3 shows the agreement between patients' preferred and perceived level of involvement in TDM for their last important treatment decision. Overall, 60% (n = 205) of participants reported that their preference for level of involvement in their last important treatment decision was met. There was moderate agreement between preferred and perceived roles in TDM (*κ* = 0.56; 95% CI, 0.34‐0.78). Of the patients whose preferences were not met, 63% (n = 85) preferred more involvement.

**Table 3:** Agreement between preferred and perceived involvement in treatment decision making (TDM) (N = 341)^a^

		Perceived				
		Patient Makes Decision	Patient Makes Decision after Seriously Considering Doctor's Opinion	Shared Responsibility	Doctor Makes Decision after Seriously Considering Patient's Opinion	Doctor Makes Decision
		N (%)	N (%)	N (%)	N (%)	N (%)
Preferred	Patient makes decision	**4 (1.2%)**	1 (0.3%)	0 (0%)	0 (0%)	0 (0%)
	Patient makes decision after seriously considering doctor's opinion	16 (4.7%)	**71 (20.8%)**	15 (4.4%)	4 (1.2%)	11 (3.2%)
	Shared responsibility	1 (0.3%)	9 (2.6%)	**63 (18.5%)**	15 (4.4%)	21 (6.2%)
	Doctor makes decision after seriously considering patient's opinion	0 (0%)	6 (1.8%)	13 (3.8%)	**22 (6.5%)**	18 (5.3%)
	Doctor makes decision	2 (0.6%)	2 (0.6%)	2 (0.6%)	0 (0%)	**45 (13.2%)**

*Note*. The proportion of participants who had concordance between perceived and preferred decision making role is in bold.

a Proportions may not sum to 100% because of rounding.

In the adjusted regression analysis, none of the analyzed patient characteristics were found to have a statistically significant association with not having preferences met.

## 4 DISCUSSION

### 4.1 Agreement between preferred and perceived involvement in TDM

In our study, approximately equal proportions of medical oncology patients preferred active, shared, or passive roles in TDM. This finding corroborates previous research^10^ and highlights a high degree of individual variability in cancer patients' preferences regarding the extent to which they would like to be involved in TDM. Sixty percent of patients had their TDM preferences met for their last important treatment decision. While the proportion of patients who perceive that they were involved in TDM at their preferred level has varied widely in past studies^9^ (31‐97%), our results are very similar to the mean concordance rates of between 58% and 61% reported for cancer treatment decisions in systematic reviews.^9^, ^10^ They also align with a recent study that found a concordance rate of 58% among a sample of over 3000 men with prostate cancer,^29^ indicating that despite increased recognition of the need to meet patients preferences for involvement in TDM, a persistent gap in care remains. Given the significant benefits associated with ensuring that patients are involved in decisions to their preferred extent, including increased satisfaction with decision making^16^, ^17^, ^18^ and reduced decisional regret,^30^ there is a need to further understand and address this important gap in cancer care. In particular, interventions that aim to increase alignment between patients' preferred and perceived roles in cancer TDM should be developed and evaluated using robust methodology.

Our finding that patients who did not have their preferences met were more likely to want more involvement in TDM also aligns with previous studies.^9^, ^10^ However, almost 40% of patients who did not have their preferences met would have preferred less involvement in TDM. The systematic review of Brom et al^9^ showed that in nine of 34 studies, cancer patients were more likely to prefer a more passive role to the one they perceived experiencing.^9^ These findings suggest that a substantial proportion of patients prefer more passive involvement in TDM than they experience, and caution against assuming that all patients prefer an active role in TDM. Some studies have shown that pushing patients to assume a more active role than they prefer can have detrimental effects, including increased anxiety^31^ and decisional regret.^15^ However, in one US study of over 800 breast cancer patients, experiencing a more active role than preferred was associated with improved social and physical well‐being and quality of life.^32^ These findings highlight the importance of informing patients about the benefits and risks of participating in TDM, when eliciting their preferred level of involvement.

### 4.2 Characteristics associated with not having TDM preferences met

In the adjusted analysis, no patient characteristics among the ones tested were significantly associated with not having TDM preferences met. This result aligns with the systematic review of Brom et al^9^ that reported mixed findings with respect to patient characteristics associated with not having TDM preferences met and recent studies that have not found an association between patient characteristics and congruence between preferred and perceived roles (eg, Hamelinck et al^33^). It was notable from this review that relatively few studies have examined characteristics associated with preferences from involvement in TDM not being met, relative to those that have examined characteristics associated with either preferred or perceived involvement in TDM. Therefore, future studies should continue to explore which patient characteristics may be associated with discordance between preferred and perceived roles in TDM. For example, given studies showing worse outcomes for cancer patients living in rural areas as compared with urban areas,^34^, ^35^ it may be worth exploring whether geographical location is associated with unmet TDM preferences. In addition, although we did not find cancer site to be significantly associated with having unmet TDM preferences, the majority of our sample (96%) was composed of patients with solid cancers. A systematic review showed that patients with hematological cancers are more likely to prefer more passive participation in TDM than patients with solid cancers.^36^ This warrants further exploration of whether the likelihood of having TDM preferences met differs for patients with hematological vs solid cancers.

### 4.3 Limitations and future directions

Nonrandom sampling of both clinics and participants is likely to have introduced some selection bias, reducing the generalizability of findings. However, there was a high consent rate, and consent bias was not evident for sex or age, indicating that participants were largely representative of the patients attending the selected clinics. Further, only 3% of the sample was aged over 80 years. Future studies should attempt to use more robust random sampling techniques to increase the representativeness of the sample.

Given that patient‐doctor interaction regarding TDM involves both parties, the lack of data on doctor characteristics is a notable limitation in our study and in much of the other published work on this topic.^9^ In light of the lack of consistency in previous studies regarding which patient characteristics are associated with discordance, it is recommended that future research includes doctor characteristics. This is often challenging given difficulties with recruiting health care providers to research studies. However, inclusion of these data is likely to assist in identifying the circumstances under which discordance occurs.

An adapted version of the original Control Preferences Scale^25^ was utilized, which may have affected the validity of the results. However, the fact that our findings were largely consistent with other similar studies suggests that the measure adaption is unlikely to have significantly impacted the findings. The self‐report nature of this measure may also have introduced biases. For example, the measure asked participants to report on a previous important treatment decision. For 30% of the sample, this occurred longer than 6 months ago, which may have introduced recall biases. Further, as preferences were measured retrospectively (ie, preferences measured after a decision took place), as opposed to prospectively (ie, preferences measured prior to a decision taking place), this may have introduced further biases. For example, the patient's past experiences in making the decision, and the consequences of that decision, may have shifted their preferences for future decision making. The systematic review of Brom et al showed that patients were more likely to report preferring a more passive role when preferences were measured retrospectively, compared with prospectively.^9^ In addition, higher concordance rates were found when preferences were measured retrospectively, compared with prospectively, suggesting that confirmation bias may have influenced patients' preferences when measured retrospectively. As decision‐making preferences may evolve throughout the course of TDM, measuring patients' preferences both before and after the decision is made using a longitudinal study design may provide valuable information about the evolution of patients' preferences. This design would also allow a more direct comparison of how the time at which preferences are measured influences concordance rates.

### 4.4 Practice implications

Our findings indicate that the process of TDM should first involve asking patients how much involvement they would like in decision making before providing assistance, to ensure that patients are having their preferences met. This could involve a simple screen to identify the patient's preferred level of involvement in TDM, including education about the risks and benefits of different levels of involvement in decision making, and the use of physician‐patient communication techniques that increase patients' comfort in expressing their needs and preferences. As preferences for involvement may also change over time, the patient's preferred level of involvement should be reassessed for each treatment decision, to ensure patients continue to have their preferences met throughout the course of their treatment planning.

### 4.5 Conclusions

Despite the growing body of literature reporting discordance in cancer patients' preferred and perceived roles in TDM, our findings show that a large proportion of cancer patients are still not having their preferences met. This highlights the need for patients' preferences for involvement in TDM to be assessed prior to the decision being made. While none of the evaluated patient characteristics were found to be associated with discordance between preferred and perceived decision making, further research is needed to identify the patients who are less likely to have their preferences for TDM met.

## FUNDING

Financial support for this study was provided entirely by a National Health and Medical Research Council Project grant (ID 1010536), a Strategic Research Partnership grant (CSR 11‐02) from Cancer Council NSW to the Newcastle Cancer Control Collaborative (New‐3C), and infrastructure funding from the Hunter Medical Research Institute (HMRI). Dr Jamie Bryant is supported by an NHMRC‐ARC Dementia Research Development Fellowship (1105809). Mariko Carey is supported by a National Health and Medical Research Council Translation of Research into Practice (TRIP) fellowship (1073031). The funding agreement ensured the authors' independence in designing the study, interpreting the data, writing, and publishing the report.

## CONFLICTS OF INTEREST

None declared.

## AUTHOR CONTRIBUTIONS

Conceptualization: Mariko Carey, Frans Henskens

Formal analysis: Elise Mansfield

Funding acquisition: Mariko Carey, Frans Henskens

Methodology: Mariko Carey, Frans Henskens

Project administration: Heidi Turon, Mariko Carey

Writing – original draft preparation: Elise Mansfield, Jamie Bryant, Mariko Carey, Heidi Turon, Frans Henskens, Alice Grady

Writing – review and editing: Elise Mansfield, Jamie Bryant, Mariko Carey, Heidi Turon, Frans Henskens, Alice Grady

## Supporting information

Data S1 Supporting Information
