# Supplementary material for: Getting the right fit: Convergence between preferred and perceived involvement in treatment decision making among medical oncology outpatients
Source: Health Sci Rep. 2018 Nov 6;2(1):e101. doi: 10.1002/hsr2.101 (PMC6346985; doi:10.1002/hsr2.101)
Supplement: Supplementary file 1 — Data S1 Supporting Information [file HSR2-2-e101-s001.docx]

**Supporting material: Survey items on involvement in treatment decision making**

| **When did you last make an important decision about your cancer treatment?**  *Please estimate the number of*  *days* ***OR*** *weeks* ***OR*** *months ago* | Days ago  **OR**  Weeks ago  **OR**  Months ago |
| --- | --- |
| **Thinking back to that treatment decision, how involved were you in making that decision?**  *Please circle one number only* | **1** I made the decision about which treatment I would receive  **2** I made the final decision about my treatment after seriously considering my doctor’s opinion  **3** Both my doctor and I shared the responsibility for deciding which treatment was best for me  **4** My doctor made the final decision about which treatment would be used, but seriously considered my opinions  **5** I left all decisions regarding my treatment to my doctor |
| **When making important treatment decisions, how involved would you like to be in making the decision?**  *Please circle one number only* | ***I prefer:***  **1** To make the decision about which treatment I will receive  **2** To make the final decision about my treatment after seriously considering my doctor’s opinion  **3** That my doctor and I share responsibility for deciding which treatment is best for me  **4** That my doctor makes the final decision about which treatment will be used, but seriously considers my opinions  **5** To leave all decisions regarding my treatment to my doctor |
